# Supplementary material for: Follow-Up Study Confirms the Presence of Gastric Cancer DNA Methylation Hallmarks in High-Risk Precursor Lesions
Source: Cancers (Basel). 2021 Jun 2;13(11):2760. doi: 10.3390/cancers13112760 (PMC8199626; doi:10.3390/cancers13112760)
Supplement: Supplementary file 1 [file cancers-13-02760-s001.zip › cancers-1213371-supplementary.pdf]

cancers MDPI

Supplementary Material: Follow-up study confirms the presence of gastric cancer DNA methylation hallmarks in high-risk precursor lesions.

Antonio Gómez, Miguel L. Pato, Luis Bujanda, Núria Sala, Osmel Companioni, Ángel Cosme, Martina Tufano, David J. Hanly, Nadia García, José Miguel Sanz-Anquela, Javier P Gisbert, Consuelo López, José Ignacio Elizalde, Miriam Cuatrecasas, Victoria Andreu[1], María José Paules, María Dolores Martín-Arranz, Luis Ortega, Elvira Poves, Jesús Barrio, María Ángeles Torres, Guillermo Muñoz, Ángel Ferrández, María José Ramírez-Lázaro, Sergio Lario, Carlos A González, Manel Esteller and María Berdasco

Table S1. Adenocarcinoma patients included in the Infinium 450K methylation study. Two samples pieces were extracted from each patient: one corresponds to the tumor and a second piece from adjacent non-tumor gastric mucosae. *n.d*; not determined; F, female; M, male.[1] Tumor size/Lymph Node involvement/Metastasis (TNM) Classification of Malignant Tumours by the International Union Against Cancer.

SAMPLE	AGE (years)	GENDER	TNM[1]	LAUREN CLASSIFICATION	DIFFERENTIATION	METASTASIS (YES/NO)	*H.pylori* infection	*H. pylori* haplotype
GC 1	86	F	T3N0	diffuse;signet-ring	poorly differentiated	no	Hp pos	VacAs2m2 CagA-
GC 2	79	F	T4N3	diffuse	poorly differentiated	yes	Hp pos	VacAs1s2m1 CagA-
GC 3	66	F	T4N2	diffuse	poorly differentiated	yes	Hp neg	--
GC 4	83	F	T3N2	diffuse	moderately differentiated	no	Hp neg	--
GC 5	69	F	T3N2	diffuse	poorly differentiated	yes	Hp neg	--
GC 6	59	M	T3N2M1	diffuse	badly differentiated	yes	Hp pos	VacAs1m1m2 Cag+
GC 7	66	M	T3N3	diffuse	badly differentiated	yes	Hp neg	--
GC 8	28	F	T3N1	diffuse	badly differentiated	yes	Hp pos	VacAs1s2m1m2 CagA+
GC 9	76	F	T4N1M0	diffuse	moderately differentiated	yes	Hp pos	VacAs1m1 CagA+
GC 10	54	F	T3N3	diffuse;signet-ring	badly differentiated	yes	Hp neg	--
GC 11	78	M	T3N3	diffuse	badly differentiated	no	Hp neg	--
GC 12	72	M	T1N0	intestinal	differentiated	no	*n.d*	--
GC 13	75	M	T2N0	intestinal	moderately differentiated	no	Hp pos	VacAs1s2m1m2 CagA+
GC 14	79	M	T3N1	intestinal	differentiated	no	Hp neg	--
GC 15	79	M	T3N0	intestinal	moderately differentiated	yes	Hp pos	VacAs1m1 CagA+
GC 16	49	M	T2N0	intestinal	poorly differentiated	no	Hp pos	VacAs1s2m2 CagA+
GC 17	87	F	T2N0	intestinal	moderately differentiated	no	Hp pos	VacAs1m1m2 CagA+
GC 18	77	M	T3N0	intestinal	moderately differentiated	yes	Hp neg	--
GC 19	78	F	T3N1	intestinal	badly differentiated	no	Hp pos	VacAs1m2 CagA+
GC 20	82	F	T2N0	intestinal	badly differentiated	no	Hp neg	--
GC 21	63	M	T3N0	intestinal	well differentiated	no	Hp neg	--
GC 22	83	F	T2N0	intestinal	well ifferentiated	no	Hp neg	--
GC 23	64	M	T2N0	intestinal	well differentiated	yes	Hp pos	VacAs1m1 CagA+
GC 24	73	M	T3N1	intestinal	differentiated	no	Hp neg	--

Table S2. Clinical features of the human subjects included in the Infinium 450K methylation study according to histological diagnoses of precursor lesions.

Sample	Age	Gender	Histological diagnoses	*H. pylori* infection	*H. pylori* haplotype
NM_1	57	Male	Normal mucosae	Hp neg	--
NM_2	64	Female	Normal mucosae	Hp neg	--
NM_3	69	Male	Normal mucosae	n.d	--
NM_4	59	Male	Normal mucosae	Hp neg	--
NM_5	59	Male	Normal mucosae	Hp neg	--
NM_6	59	Male	Normal mucosae	Hp neg	--
NM_7	69	Female	Normal mucosae	Hp neg	--
NM_8	82	Female	Normal mucosae	Hp neg	--
NM_9	65	Female	Normal mucosae	Hp pos	VacAs2m2 CagA-
NM_10	60	Female	Normal mucosae	Hp neg	--

NAG_1	67	Female	Non-atrophic gastritis	Hp pos	VacAs1s2m1m2 CagA-
NAG_2	75	Male	Non-atrophic gastritis	Hp pos	VacAs1m1 CagA+
NAG_3	58	Female	Non-atrophic gastritis	Hp pos	VacAs1s2m1 CagA-
NAG_4	48	Male	Non-atrophic gastritis	Hp pos	VacAs1s2m1m2 CagA-
NAG_5	65	Female	Non-atrophic gastritis	Hp pos	VacAs1s2m1m2 CagA-
NAG_6	69	Male	Non-atrophic gastritis	Hp pos	VacAs1m1 CagA-
NAG_7	69	Male	Non-atrophic gastritis	Hp pos	VacAs1s2m1 CagA+
NAG_8	66	Female	Non-atrophic gastritis	Hp pos	VacAs1m1 CagA+
NAG_9	69	Female	Non-atrophic gastritis	Hp neg	--
NAG_10	59	Male	Non-atrophic gastritis	Hp pos	VacAs1m1 CagA-
CAG_1	72	Female	Multifocal chronic atrophic gastritis	n.d	--
CAG_2	63	Female	Multifocal chronic atrophic gastritis	n.d	--
CAG_3	58	Female	Multifocal chronic atrophic gastritis	Hp pos	VacAs1s2m1 CagA-
CAG_4	58	Female	Multifocal chronic atrophic gastritis	Hp neg	--
CAG_5	82	Female	Multifocal chronic atrophic gastritis	Hp neg	--
CAG_6	77	Male	Multifocal chronic atrophic gastritis	Hp neg	--
CAG_7	66	Male	Multifocal chronic atrophic gastritis	Hp neg	--
CAG_8	60	Male	Multifocal chronic atrophic gastritis	Hp pos	VacAs2m2 CagA-
CAG_9	49	Female	Multifocal chronic atrophic gastritis	Hp pos	VacAs2m1 CagA-
CAG_10	49	Female	Multifocal chronic atrophic gastritis	Hp neg	--
CAG_11	77	Female	Multifocal chronic atrophic gastritis	Hp pos	VacAs1s2m1 CagA+
CAG_12	77	Female	Multifocal chronic atrophic gastritis	Hp pos	VacAs1m1 CagA+
CAG_13	61	Male	Multifocal chronic atrophic gastritis	Hp neg	--
CIM_1	57	Male	Complete intestinal metaplasia	Hp neg	--
CIM_2	76	Male	Complete intestinal metaplasia	Hp neg	--
CIM_3	76	Male	Complete intestinal metaplasia	n.d	--
CIM_4	69	Male	Complete intestinal metaplasia	n.d	--
CIM_5	73	Female	Complete intestinal metaplasia	Hp pos	VacAs1m1 CagA-
CIM_6	57	Female	Complete intestinal metaplasia	Hp pos	VacAs1s2m1 CagA+
CIM_7	75	Male	Complete intestinal metaplasia	n.d	--
CIM_8	52	Female	Complete intestinal metaplasia	Hp pos	VacAs1s2m2 CagA+
CIM_9	67	Female	Complete intestinal metaplasia	Hp neg	--
CIM_10	77	Male	Complete intestinal metaplasia	Hp pos	VacAs1m1 CagA-
CIM_11	70	Female	Complete intestinal metaplasia	Hp neg	--
CIM_12	70	Female	Complete intestinal metaplasia	Hp neg	--
IIM_1	64	Female	Incomplete intestinal metaplasia	Hp pos	VacAs1m2 CagA+
IIM_2	57	Female	Incomplete intestinal metaplasia	Hp pos	VacAs1m1 CagA+
IIM_3	75	Male	Incomplete intestinal metaplasia	Hp pos	VacAs1m1 CagA-
IIM_4	71	Male	Incomplete intestinal metaplasia	Hp neg	--
IIM_5	68	Female	Incomplete intestinal metaplasia	Hp pos	VacAs1m1 CagA+
IIM_6	68	Female	Incomplete intestinal metaplasia	Hp pos	VacAs1m1 CagA+
IIM_7	68	Female	Incomplete intestinal metaplasia	Hp pos	VacAs1m1 CagA+
IIM_8	67	Female	Incomplete intestinal metaplasia	Hp neg	--
IIM_9	67	Female	Incomplete intestinal metaplasia	Hp neg	--
IIM_10	78	Female	Incomplete intestinal metaplasia	Hp pos	VacAs2m2 CagA-
IIM_11	82	Female	Incomplete intestinal metaplasia	Hp pos	VacAs2m2 CagA-
IIM_12	66	Male	Incomplete intestinal metaplasia	Hp neg	--
IIM_13	48	Male	Incomplete intestinal metaplasia	Hp pos	VacAs1m1 CagA-
IIM_14	60	Female	Incomplete intestinal metaplasia	Hp neg	--
IIM_15	74	Female	Incomplete intestinal metaplasia	Hp neg	--
IIM_16	74	Female	Incomplete intestinal metaplasia	Hp neg	--

IIM_17	81	Female	Incomplete intestinal metaplasia	Hp pos	VacAs1m1 CagA+
IIM_18	60	Male	Incomplete intestinal metaplasia	Hp pos	VacAs1m1 CagA-
IIM_19	60	Male	Incomplete intestinal metaplasia	Hp pos	VacAs1m1 CagA-

Table S3. Representative (top-20) differentially methylated CpGs (DMCpG) obtained after statistical comparison of intestinal and diffuse type of gastric cancer and normal adjacent tissues.

**Representative DMCpG in DIFFUSE subtype of gastric cancer**				
**Target ID**	**pval**	**UCSC_Name**	**Relation_to_Island**	**Methylation level in cancer tissue**
cg17994840	1,17E-09	INPP5A	OpenSea	Hypermethylated
cg07532183	1,42E-09		OpenSea	Hypermethylated
cg14854315	1,52E-09	SSH1	OpenSea	Hypermethylated
cg19848599	2,46E-09	SLC22A4	OpenSea	Hypermethylated
cg08396985	2,10E-08		OpenSea	Hypermethylated
cg04570362	5,64E-08	CLSTN1	OpenSea	Hypermethylated
cg22518433	8,86E-08	FUT1	Island	Hypermethylated
cg15284457	1,30E-07		OpenSea	Hypermethylated
cg24600221	4,53E-07	IGF1R	OpenSea	Hypermethylated
cg00920668	6,55E-07	LOC148696	OpenSea	Hypermethylated
cg25947619	6,55E-07	AKAP13	OpenSea	Hypermethylated
cg23024775	6,95E-07	BRI3	OpenSea	Hypermethylated
cg01961086	9,53E-07	PRDM16	OpenSea	Hypermethylated
cg09768859	9,73E-07	INPP5A	OpenSea	Hypermethylated
cg13305444	1,87E-06	ENAH	N_Shelf	Hypermethylated
cg06601579	4,28E-06		OpenSea	Hypermethylated
cg06330323	6,17E-06	TSC2	N_Shore	Hypermethylated
cg18125573	0,000117111	RARA	OpenSea	Hypermethylated
cg18372896	0,000229151	JDP2	S_Shore	Hypermethylated
cg18811130	0,002960529	MGMT	S_Shelf	Hypermethylated
cg15187223	4,05E-09	RASA3	N_Shelf	Hypomethylated
cg17266282	5,45E-09		OpenSea	Hypomethylated
cg23839180	5,67E-09	FAM49A	OpenSea	Hypomethylated
cg10576245	3,69E-08	PCNXL2	OpenSea	Hypomethylated
cg17441401	3,72E-08	LRP8	S_Shore	Hypomethylated
cg22359781	4,43E-08		OpenSea	Hypomethylated
cg04786142	5,55E-08		OpenSea	Hypomethylated
cg04456219	9,64E-08		OpenSea	Hypomethylated
cg02263377	9,97E-08	ADSSL1	OpenSea	Hypomethylated
cg14094027	1,23E-07	PXN	OpenSea	Hypomethylated
cg16734433	5,78E-07		N_Shore	Hypomethylated
cg07436694	1,82E-06	METTL9	OpenSea	Hypomethylated
cg18847089	4,29E-06	PRKAR1B	Island	Hypomethylated
cg21422164	8,13E-06	RASA3	N_Shelf	Hypomethylated
cg16499677	0,000165294	C14orf37	OpenSea	Hypomethylated
cg10423607	4,99E-04	CPA6	OpenSea	Hypomethylated
cg19486070	0,00127115	KIAA1908	OpenSea	Hypomethylated
cg27341866	0,001755898	C19orf35	Island	Hypomethylated
cg23460250	0,005446902		OpenSea	Hypomethylated
cg26858540	0,007444221	ZNF787	S_Shelf	Hypomethylated
**Representative DMCpG in INTESTINAL subtype of gastric cancer**				
**Target ID**	**pval**	**UCSC_Name**	**Relation_to_Island**	**Methylation level in cancer tissue**
cg23216292	1,48E-09	ZNF85	OpenSea	Hypermethylated
cg10146929	2,32E-09	HIST1H1A	N_Shelf	Hypermethylated

cg10868817	3,12E-09	ZFPM2	Island	Hypermethylated
cg09989996	3,37E-09		OpenSea	Hypermethylated
cg12367389	4,78E-09	HTR2A	OpenSea	Hypermethylated
cg18158151	5,26E-09	C2orf74	OpenSea	Hypermethylated
cg24862510	2,94E-08		Island	Hypermethylated
cg25947619	5,95E-08	AKAP13	OpenSea	Hypermethylated
cg24239882	0,00010425	ST8SIA1	Island	Hypermethylated
cg05037927	0,000124686	C2orf74	OpenSea	Hypermethylated
cg03146625	0,000154462	HOXC4	S_Shore	Hypermethylated
cg26407571	0,000234931	FLJ12825	Island	Hypermethylated
cg24757310	0,000287479	C2orf74	OpenSea	Hypermethylated
cg13879483	0,000425155	USP44	Island	Hypermethylated
cg08048222	0,000489101	ZNF671	Island	Hypermethylated
cg16328106	0,00073697	C2orf74	OpenSea	Hypermethylated
cg15779837	0,000884686	GRIN2D	Island	Hypermethylated
cg09489445	0,001035597	ZNF788	Island	Hypermethylated
cg14103680	0,001888117		Island	Hypermethylated
cg04605980	0,00326941		OpenSea	Hypermethylated
cg20482390	1,76E-09	C20orf195	N_Shore	Hypomethylated
cg24622143	3,99E-09	RASA3	Island	Hypomethylated
cg10938046	8,50E-09	C6orf223	N_Shore	Hypomethylated
cg16001418	1,46E-08	HCST	S_Shore	Hypomethylated
cg18692507	0,000108057		N_Shelf	Hypomethylated
cg01719405	0,000171742		OpenSea	Hypomethylated
cg04436383	0,000220504	SLC10A6	OpenSea	Hypomethylated
cg23739746	0,000271604		OpenSea	Hypomethylated
cg24878483	0,000278317	LRP1B	OpenSea	Hypomethylated
cg02954324	0,000302095		N_Shelf	Hypomethylated
cg09981464	0,000313051	ZCCHC14	Island	Hypomethylated
cg01432520	0,00042542	ESRRG	OpenSea	Hypomethylated
cg20141733	0,000790828		OpenSea	Hypomethylated
cg19982609	0,001386191		OpenSea	Hypomethylated
cg13816734	0,001410061	GPC5	OpenSea	Hypomethylated
cg24339273	0,001888991		OpenSea	Hypomethylated
cg23369601	0,00214212	NSUN2	N_Shore	Hypomethylated
cg08324862	0,002392503		OpenSea	Hypomethylated
cg17660833	0,003403534	HRH1	OpenSea	Hypomethylated
cg08357895	0,004340688		N_Shore	Hypomethylated

Table S4. Representative (top-20) differentially methylated CpGs in intestinal metaplasia and normal mucosa.

Representative DMCpG in INTESTINAL METAPLASIA				
**Target ID**	**pval**	**UCSC_Name**	**Relation_to_Island**	**Methylation level in cancer tissue**
cg01808171	1,54E-09	ZNF626	OpenSea	Hypermethylated
cg10108296	4,66E-09	TRIM15	N_Shore	Hypermethylated
cg18573842	6,75E-009	TRIM15	N_Shore	Hypermethylated
cg05341539	1,03E-08	AKAP13	OpenSea	Hypermethylated
cg00030508	1,21E-08	TRIM15	N_Shore	Hypermethylated
cg05354921	1,59E-04	PRDM16	N_Shelf	Hypermethylated
cg13000649	2,46E-04	RAI1	Island	Hypermethylated
cg10119075	3,22E-04	ITIH5	OpenSea	Hypermethylated
cg10656016	3,53E-04	PLCXD2	OpenSea	Hypermethylated
cg25291138	3,54E-04	SFMBT2	Island	Hypermethylated
cg07209071	3,62E-04	PRDM16	N_Shelf	Hypermethylated
cg12948621	4,28E-04	HKR1	Island	Hypermethylated
cg00486998	5,58E-04	MECOM	OpenSea	Hypermethylated
cg26309134	7,44E-04	ZNF542; ZNF542; ZNF542; ZNF542	Island	Hypermethylated
cg19418951	8,24E-04	MAP3K13	OpenSea	Hypermethylated
cg10311806	8,40E-04	ITIH5	OpenSea	Hypermethylated
cg04145134	1,01E-03	BCL11B	OpenSea	Hypermethylated
cg16501625	0,002343055		OpenSea	Hypermethylated
cg11258943	4,10E-03	FOXE1	Island	Hypermethylated
cg18757468	0,00527511	EHBP1L1	N_Shore	Hypermethylated
cg23271915	1,13E-009		OpenSea	Hypomethylated
cg08282819	1,60E-09	IL21R	OpenSea	Hypomethylated
cg02389317	2,85E-09		OpenSea	Hypomethylated
cg06936290	3,17E-09		OpenSea	Hypomethylated
cg15058210	4,04E-09	HDAC4	Island	Hypomethylated
cg05032059	4,92E-009		OpenSea	Hypomethylated
cg03000596	7,10E-09	LAT2	OpenSea	Hypomethylated
cg15978561	1,25E-08	HDAC4	Island	Hypomethylated
cg05870586	1,73E-08	HDAC4	Island	Hypomethylated
cg24722577	3,24E-08		OpenSea	Hypomethylated
cg02239453	4,98E-07	FBXO3;FBXO3	S_Shore	Hypomethylated
cg02983090	5,27E-07	IL21R	OpenSea	Hypomethylated
cg23907053	6,01E-07	RAB3IP	OpenSea	Hypomethylated
cg01492656	6,31E-07	N4BP2	N_Shore	Hypomethylated
cg15635368	3,08E-005	INPP1	N_Shore	Hypomethylated
cg23464619	1,34E-04		OpenSea	Hypomethylated
cg15978565	0,000204362	LPIN1	OpenSea	Hypomethylated
cg11201447	3,53E-04	MIR1204	S_Shore	Hypomethylated
cg03183540	3,61E-04		OpenSea	Hypomethylated
cg01649611	0,000369131	THADA	OpenSea	Hypomethylated

Table S5. Representative (top-20) differentially methylated CpGs depending on *Helicobacter pylori* infection in multifocal chronic atrophic gastritis.

Representative DMCpG associated with *Hp* infection				
**Target ID**	**pval**	**UCSC_Name**	**Relation_to_Island**	**Methylation level in infected samples**
cg04503968	4,77E-03	PRDM6	N_Shore	Hypermethylated
cg01958086	4,74E-03	MEIS2	N_Shore	Hypermethylated
cg10074409	4,63E-03	IRF6	Island	Hypermethylated
cg04911005	4,50E-03	LXN	OpenSea	Hypermethylated
cg01249187	4,37E-03	CACNA1H	Island	Hypermethylated
cg11362604	4,32E-03	MEIS2	N_Shore	Hypermethylated
cg07100771	4,20E-03	UCP1	Island	Hypermethylated
cg15195321	0,003864981	HAND2	Island	Hypermethylated
cg24357619	3,55E-03	MEIS2	N_Shore	Hypermethylated
cg14129931	3,47E-03		N_Shore	Hypermethylated
cg22532843	3,40E-03		N_Shore	Hypermethylated
cg02224994	3,02E-03	C14orf64	OpenSea	Hypermethylated
cg19302996	2,82E-03	UNK	N_Shore	Hypermethylated
cg14906455	0,002716172		Island	Hypermethylated
cg19766460	2,45E-03	UMODL1	S_Shore	Hypermethylated
cg11462865	1,94E-03	KRT19	Island	Hypermethylated
cg06622408	1,78E-03		Island	Hypermethylated
cg00817464	1,64E-03	XPNPEP1	OpenSea	Hypermethylated
cg10135260	1,26E-03		Island	Hypermethylated
cg11849717	0,0010563	EGFR	Island	Hypermethylated
cg21834248	6,96E-08	TAF1A	S_Shore	Hypomethylated
cg25604067	0,000901799	ST3GAL4	Island	Hypomethylated
cg16673477	1,07E-03	CRTC1	S_Shore	Hypomethylated
cg10048349	0,001359094	CRYAB	OpenSea	Hypomethylated
cg01758314	1,59E-03	ALOX12P2	N_Shore	Hypomethylated
cg10387769	1,66E-03	MIR574	Island	Hypomethylated
cg08903855	1,71E-03		OpenSea	Hypomethylated
cg12109838	1,72E-03	NHEDC2	N_Shore	Hypomethylated
cg07136873	1,84E-03		Island	Hypomethylated
cg15100135	2,41E-03	INCA1	S_Shore	Hypomethylated
cg21917728	0,002933747		N_Shore	Hypomethylated
cg06894687	2,94E-03	QSOX1	N_Shore	Hypomethylated
cg26645655	0,003531414		OpenSea	Hypomethylated
cg21992238	3,76E-03		N_Shore	Hypomethylated
cg00864474	4,11E-03	NTN1	Island	Hypomethylated
cg02587316	4,20E-03	ZNF529	Island	Hypomethylated
cg01234063	4,45E-03	ST3GAL4	Island	Hypomethylated
cg23589617	4,58E-03		Island	Hypomethylated
cg24382276	0,004829532		OpenSea	Hypomethylated
cg12762733	4,84E-03		N_Shore	Hypomethylated

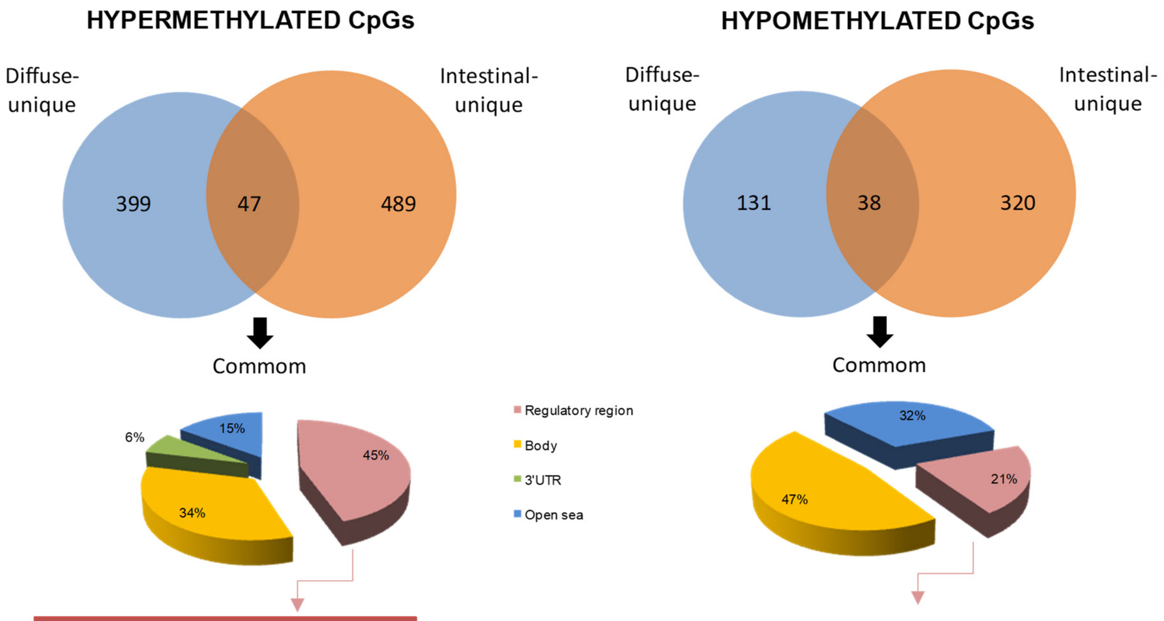

TargetID	Gene	Genomic location	CpG density
cg01287342	TTPA	TSS1500	S_Shore
cg01423964	KCNA3	1stExon	Island
cg07808555	KCNA3	TSS200	Island
cg11595545	KCNA3	1stExon	Island
cg02970696	ZNF148	5'UTR	Island
cg05249988	ZNF132	TSS200	Island
cg05963618	ADRA1B	TSS1500	Island
cg06392169	IRF4	5'UTR	Island
cg17228900	IRF4	1stExon	Island
cg26983469	VIM	5'UTR	Island
cg11973177	VIM	5'UTR	Island
cg12417071	CCNA1	TSS1500	N_Shore
cg14091954	FLT3	TSS200	Island
cg18372896	JDP2	5'UTR	S_Shore
cg19940077	GPR25	1stExon	Island
cg21521683	ST8SIA1	TSS200	Island
cg22535307	FBN1	TSS1500	Island
cg24239882	ST8SIA1	TSS1500	Island

TargetID	Gene	Genomic location	CpG density
cg01291854	ELK3	5'UTR	n.d
cg02263377	ADSSL1	TSS1500	n.d
cg03003434	SYTL3	Body	n.d
cg18419977	SLC22A18	TSS1500	N_Shelf
cg22888958	CREB5	5'UTR	n.d
cg23264429	STAMBPL1	5'UTR	S_Shore
cg23839180	FAM49A	5'UTR	n.d
cg26450106	CST7	TSS1500	n.d
cg27051318	CHRNA6	TSS200	n.d

Figure S1. Venn diagram of hypermethylated (left) and hypomethylated (right) CpGs in tumoral tissues when intestinal and diffuse subtypes of gastric cancer were compared. The genomic context (regulatory region, body, 3'UTR and intergenic) for the common cancer signature is indicated as well as the top differentially methylated sequences into regulatory regions of known genes.

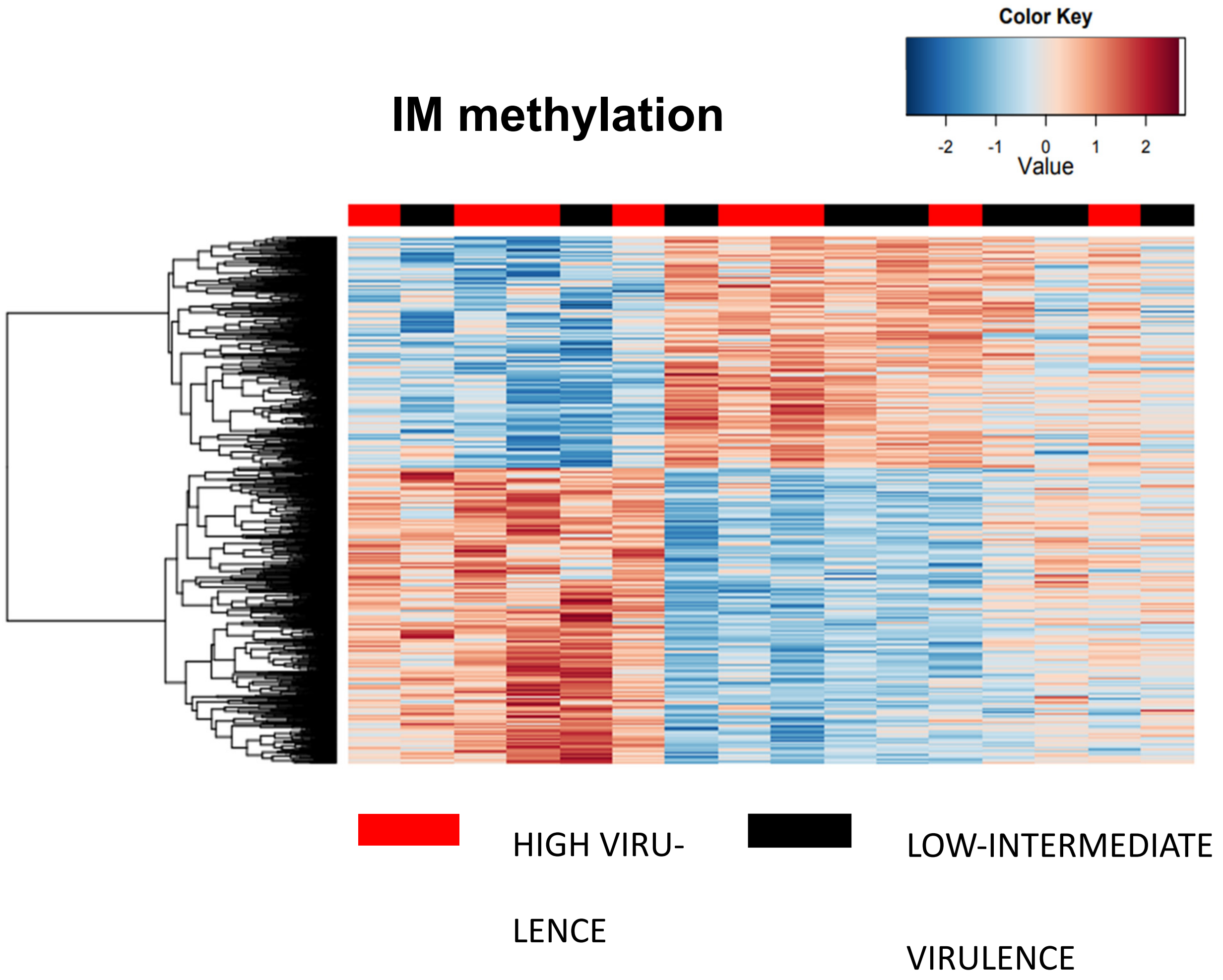

Figure S2. Supervised clustering of β-values from Infinium 450 methylation array in intestinal metaplasia (IM) with *H. pylori* infection. Samples were categorized as low-intermediate virulence (LIV) or high virulence (HV) haplotypes. Red and blue colors indicate high and low levels of DNA methylation, respectively.

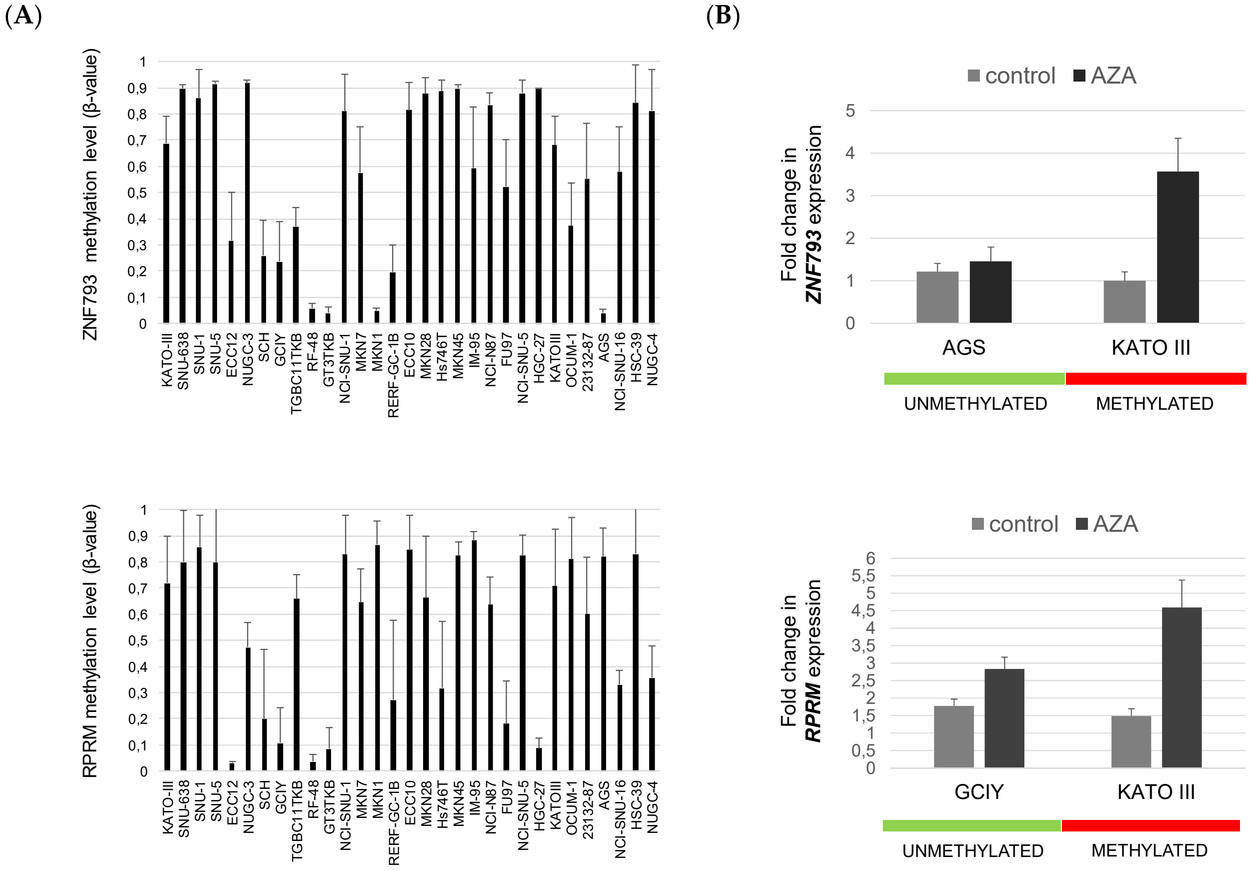

Figure S3. DNA methylation and gene expression of ZNF793 and RPRM genes in gastric cancer cell lines. **(A)** CpG methylation values of *ZNF793* and *RPRM* genes in a panel of 31 gastric cancer cell lines. Methylation values represent the average of β-value obtained for the three cg probes in the methylation array. **(B)** Expression levels of *ZNF793* and *RPRM* genes before and after treatment with the demethylating agent 5-aza-2′-deoxycytidine (AZA) in gastric cell lines.

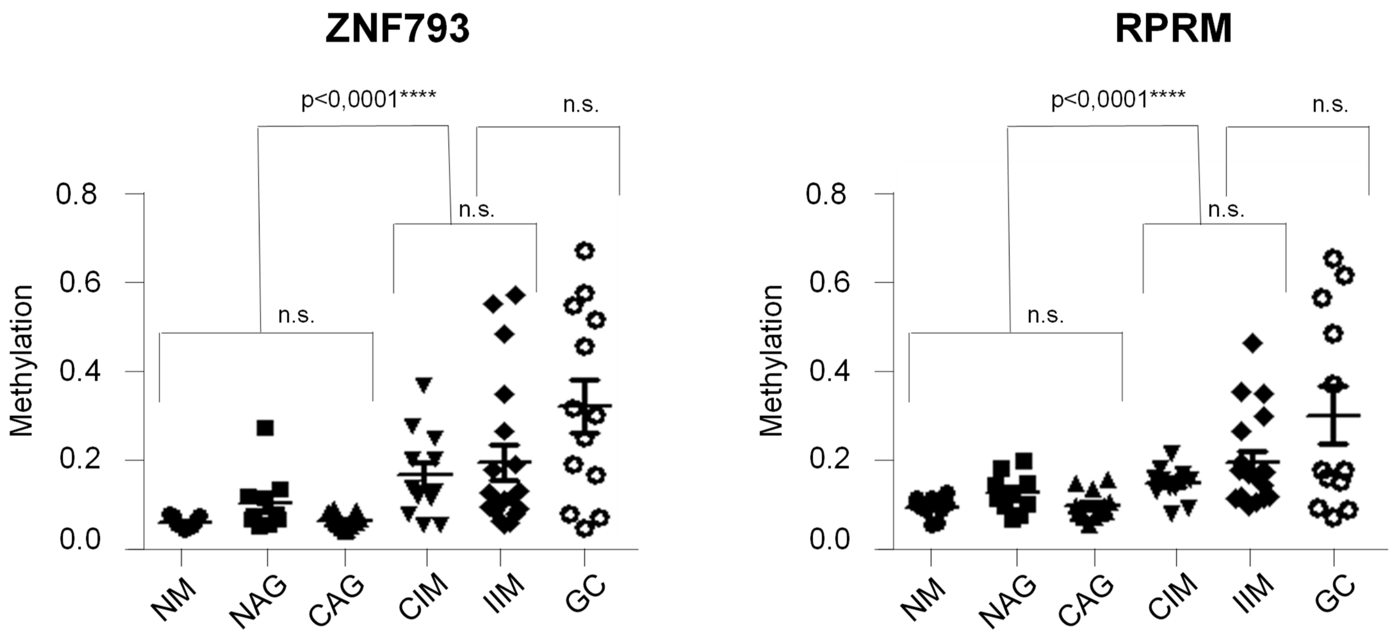

Figure S4. Methylation level of *ZNF793* and RPRM genes in normal mucosa, precursor lesions and intestinal type of gastric cancer. Methylation data are corresponded to β-values from array (cg02711801 for ZNF793 and cg00341742 for RPRM). Horizontal bars represent the median of each sample group (p<0.0001 determined by a two-tailed Wilcoxon-Mann-Whitney test). CAG, multifocal chronic atrophic gastritis; IM, intestinal metaplasia; NAG, non-atrophic gastritis; NM, normal mucosa.

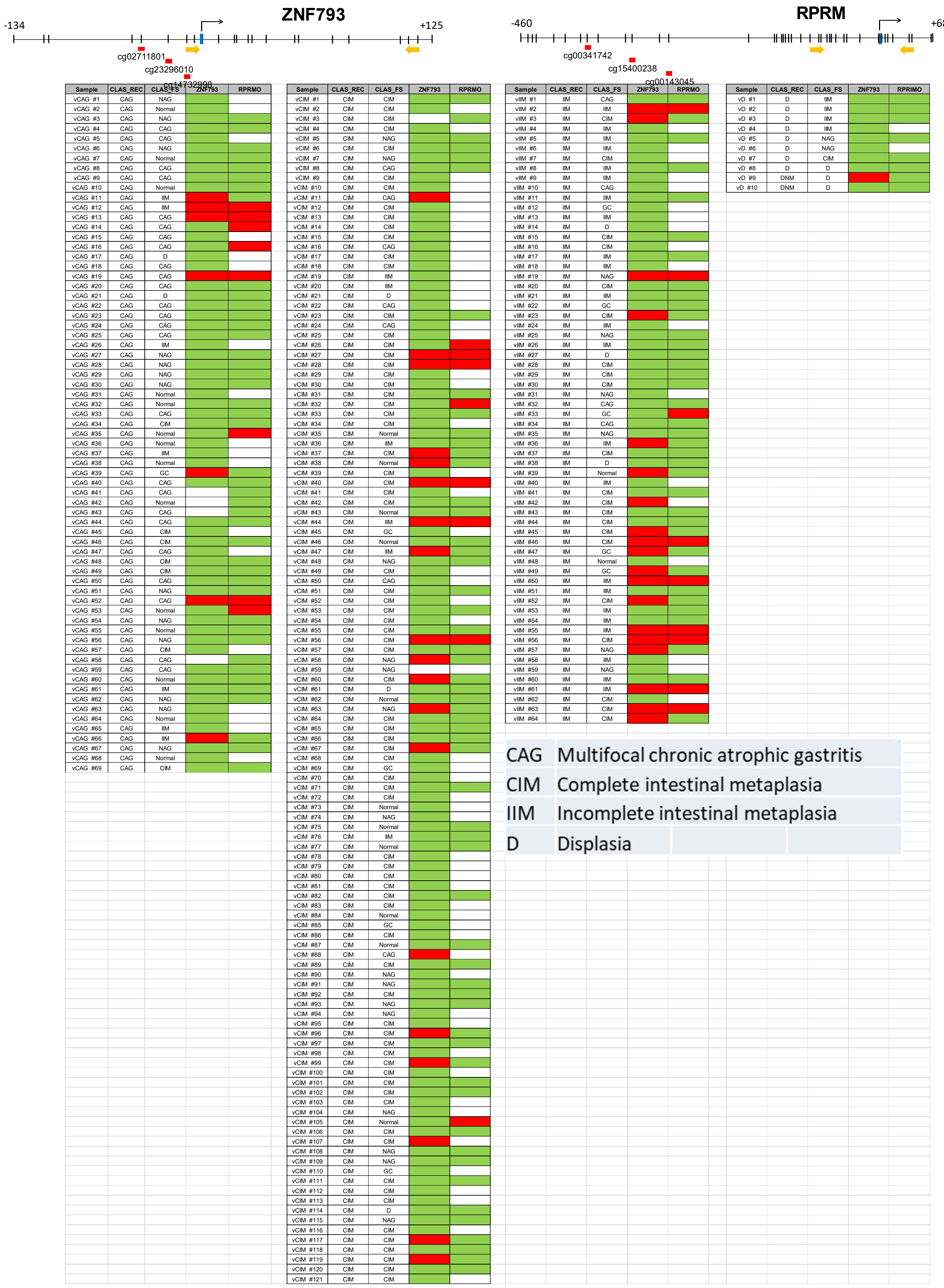

Figure S5. Summary of the methylation status of ZNF793 and RPRM genes validated by Methylation-specific promoter (MSP) in an independent cohort of precursor lesions. Upper panel, Schematic representation of the regulatory regions studied for ZNF793 (left) and RPRM (right) genes. Red squares represent the cg probes included in the methylation array.

Blue vertical bar and arrow indicate the transcription start site. Yellow arrows indicate the oligonucleotides selected for MSP studies. Lower panel, Results from 258 and 169 samples are shown for ZNF793 and RPRM genes, respectively. The diagnosis (determined by pathology analysis) at the time of recruitment and at the follow up after 12 years are indicated. Red box, methylated; green box, unmethylated.
